# Navx-guided Cryoablation of Atrial Tachycardia Inside the Left Atrial Appendage

**Published:** 2011-02-07

**Authors:** Claudio Pandozi, Marco Galeazzi, Carlo Lavalle, Sabina Ficili, Maurizio Russo, Massimo Santini

**Affiliations:** Dipartimento Cardiovascolare, Ospedale San Filippo Neri, Rome, Italy

**Keywords:** Cryoablation, Left atrial appendage, Electroanatomical mapping

## Abstract

Radiofrequency ablation procedures inside the left atrial appendage (LAA) are likely to involve dangerous complications because of a high thrombogenic effect. Cryoablation procedures are supposed to be safer. We describe two cases of successful cryoablation procedures. Two NavX-guided cryoablations of permanent focal atrial arrhythmias arising from the LAA were performed. Left atrial reconstruction and mapping allowed the zone of the earliest atrial potential to be recorded; the entire course of the ablation catheter was monitored. The arrhythmias were successfully ablated; no thrombotic complications were observed.

## Introduction

The left atrial appendage (LAA) is considered an important thrombogenic site due to its irregular shape and its tendency to get stunned during atrial arrhythmias, especially after successful cardioversion [1]. Radiofrequency (RF) ablation is capable to generate a thrombogenic setting [2]: whenever such procedure is going to be performed inside the cardiac chambers, it should be accompanied by a continuous checking of a valid anticoagulation level. Moreover, a high risk to generate steam pops and to perforate the atrial wall has been reported during RF delivery, especially in the LAA [3-4]. For these reasons some concerns exist as regard to the possibility to perform successful and safe RF ablation procedures inside the LAA. Nevertheless, such procedure has sometimes been performed [5], even by using more invasive tools like thoracoscopy [6]. On the other hand, the cryoablation is considered a safer technique because of the greater stability of the ablation catheter and the particular source of energy utilized to generate tissue damage. Such procedure is therefore less thrombogenic and could result very useful in certain arrhythmogenic settings. We describe two cases of left sided focal atrial rhythm originating from the LAA, which were successfully and safely treated by the cryoablation.

## Patient 1

**Figure 1:**
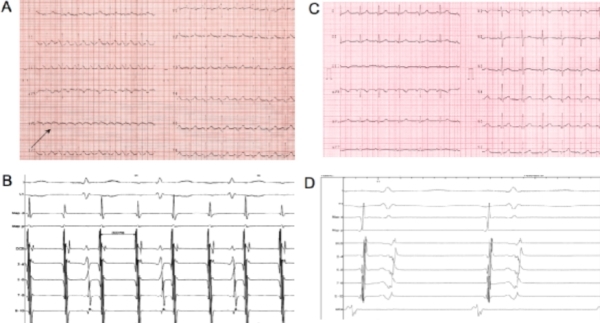
Basal ECG (A): atrial tachycardia with 2:1 conduction is detectable. The atrial waves morphology (negative in lateral leads - see black arrow - and positive in V1) involves the likely diagnosis of left sided atrial tachycardia.  Intracardiac electrograms (B) show left atrial tachycardia (cycle length 300 msec) with 2:1 conduction to the ventricles; the distal dipole of the catheter positioned inside the coronary sinus is the first one to be recording atrial signals, thus suggesting a left lateral origin of the tachycardia. The registration speed is 100 mm/sec. Post-procedural surface ECG (C): sinus rhythm is visible.  Intracardiac electrograms (D) confirm the earliest atrial activation to be at the level of the high right atrium (HRA).  Map d = distal dipole of the ablation catheter. Map p = proximal dipole of the ablation catheter. DCS = distal dipole of the catheter in coronary sinus. HRA = catheter positioned at the level of the high right atrium

A 20-year-old woman came to our attention referred by her general practitioner for the persistence of high basal heart rate in the absence of severe symptoms. The patient was complaining several occasions of self-perception of strong and accelerated heart beats, even in the absence of physical efforts. She was not taking drugs. No history of syncope or dizziness was collected. Physical examination was normal. Basal ECG (Figure 1A) showed left sided atrial tachycardia and 2:1 relation with normal QRS complexes; atrial rate was 200/min. Thyroid function was normal. Chest x-ray was normal. Basal trans-thoracic echocardiogram showed normal parameters. Electrical cardioversion was ineffective. A diagnostic electrophysiologic study was then performed: the intracardiac electrograms confirmed the presence of a left sided atrial tachycardia (cycle length 300 msec) with 2:1 conduction to the ventricles (Figure 1B). This tachycardia did not respond to adenosine bolus or beta-blocker administration. A left atrial electrical mapping was then performed, facilitated by the presence of a patent oval foramen: the focus of the tachycardia was detected inside the LAA. For technical reasons it was impossible to perform a direct cryoablation of the arrhythmia, so another procedure guided by electroanatomical mapping (EnSite NavX system; Endocardial Solutions, St Jude Medical Inc., St Paul, MN, USA) was scheduled. Geometry of the left atrium, of the pulmonary veins and of the LAA was reconstructed by the roving ablation catheter (Cool Path 4 mm irrigated, Irvine Biomedical Inc. St. Jude Medical). Rapid sequential points collection and final left atrial geometry was obtained in less than 15 minutes.

**Figure 2:**
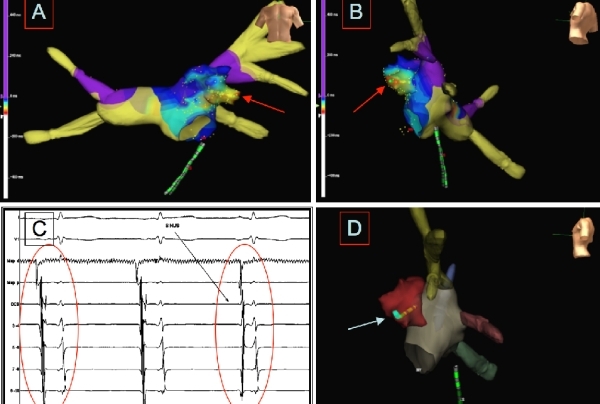
Electrophysiologic study with electroanatomical reconstruction of the left atrium (A-B). Left atrial mapping by NavX tool has confirmed the LAA to host the tachycardia source. A 4 mm Cryocath catheter has been advanced inside the LAA up to the site of the earliest activation signal (red arrow), at the level of the superior LAA recess. It is possible to appreciate two different aspects of the left atrial electroanatomical mapping.  Cardiac electrograms (C). D1, V1, the two dipoles of the ablation catheter and the five dipoles of the coronary sinus catheter (from the distal to the proximal one) are shown. Starting of the cryomapping phase (-30ºC) at the level of the superior LAA recess causes nearly immediate interruption of the tachycardia, with sudden changing of the activation sequence (from proximal to distal) along the dipoles of the catheter positioned inside the coronary sinus (black arrow). Baseline artefacts are visible at the level of the distal dipole of the Cryocath; these artefacts are generated during the cryomapping phase. The registration speed is 100 mm/sec.  Cryoablation monitoring by NavX (D). The exact location of the cryolesion is indicated by the white arrow. Map d = distal dipole of the ablation catheter. Map p = proximal dipole of the ablation catheter. DCS = distal dipole of the catheter in the coronary sinus.

Left atrial activation mapping documented LAA to host the tachycardia source. A 4-mm Cryocath catheter (Freezor, CryoCath Tecnologies Inc, Montreal, Quebec, Canada) was then advanced inside the LAA and guided up to the site of the earliest activation signal, at the level of the superior LAA lobe (Figure 2A-B). The NavX system allowed precise navigation of the Cryocath catheter and during ablation assessment of catheter stability without the use of fluoroscopy. Starting of the cryomapping procedure (-30ºC) caused interruption of the tachycardia, preceded by transient slowing of the cycle length, about 4 seconds after the achievement of the threshold temperature (Figure 2C-D). Two consecutive 8-minute sequences of cryoablation (-80ºC) were then delivered. At the end of the procedure, the 12-lead ECG and the intracardiac electrograms showed a normal sinus rhythm (Figure 1C-D). No electrical isolation of the LAA was detected. No atrial tachycardia was further inducible. The patient was administered continuous intravenous heparin throughout the procedure in the left atrium, aimed to keep an activated clotting time >250 seconds. Such infusion was continued for 24 hours after the procedure. Meanwhile oral anticoagulation therapy was started, in order to avoid possible generation of thrombi related to the atrial stunning [7]. The day after, the patient underwent transesophageal echocardiogram (TEE) showing normal LAA function, with normal flow velocities and complete absence of thrombi. She was discharged on anticoagulation therapy aimed to keep an INR value >2 for one month. We adopted such therapeutic behaviour as an additional safety measure. Further ECG and clinical controls confirmed the persistence of sinus rhythm. The patient is still known to be asymptomatic and in sinus rhythm after 3 years of clinical follow up, in complete wash out from any antiarrhytmic drug.

## Patient 2

**Figure 3:**
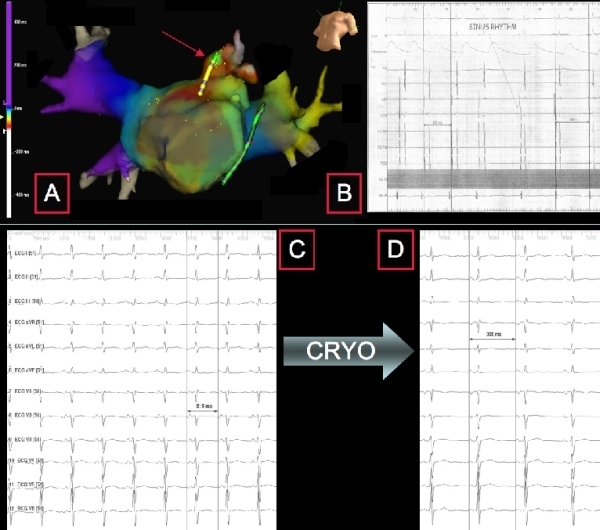
Left atrial NavX mapping (A). The LAA has been confirmed to host the ectopic source of the basal rhythm. A 4 mm Cryocath catheter has been advanced inside LAA up to the site of the earliest activation signal (red arrow), at the level of the superior LAA recess.  Intracardiac electrogram during ablation (B). Also in this case a sudden change in the activation sequence of the atrium is recorded as soon as a normal sinus rhythm is restored. The distal dipole of the His bundle recording catheter (HBD) shows now the first atrial potential as compared to the dipoles of the catheter positioned inside the coronary sinus (DCS, CS 5-6, CS 7-8, PCS) where the atrium was the first one to be activated in course of the atrial ectopic rhythm. Baseline artefacts are visible at the level of the distal dipole of the Cryocath; these artefacts are generated during the cryomapping phase. The registration speed is 33 mm/sec. Basal ECG (C). An atrial rhythm with 1:1 conduction is detectable. The atrial waves morphology (negative in the lateral leads and positive in V1) is consistent with the diagnosis of a left atrial rhythm (cycle length 615 msec). The registration speed is 50 mm/sec. Final ECG (D). As soon as the cryomapping phase (-30ºC) beginned at the level of the earliest activation signal in the LAA, the ectopic rhythm turned into sinus rhythm (cycle length 956 msec). The registration speed is 50 mm/sec. HBD = distal dipole of the catheter positioned on the His bundle. HBP = proximal dipole of the catheter positioned on the His bundle. DCS = distal dipole of the catheter in the coronary sinus. PCS = proximal dipole of the catheter in the coronary sinus. ABL D = distal dipole of the ablation catheter. ABL P = proximal dipole of the ablation catheter.

A 69-year-old man came to our attention complaining several episodes of palpitations in the absence of physical efforts. He was not taking drugs. Physical examination, basal ECG, trans-thoracic echocardiogram, chest x-ray and thyroid function were normal. A 24-hour ECG Holter recording showed one symptomatic episode of narrow QRS tachycardia (rate 110 bpm) with evidence of PR=RP. During the electrophysiologic study, the infusion of isoprenaline induced a left atrial rhythm with 1:1 relation with normal QRS complexes; the atrial rate was about 100/min. Intracardiac electrograms confirmed the presence of a left atrial tachycardia (cycle length 615 msec, see Figure 3C). This tachycardia did not respond to adenosine bolus or beta-blocker administration. An ablation procedure guided by NavX electroanatomical mapping was then scheduled, after the execution of a cardiac CT-scan. We decided to rely on this imaging technique to better define the anatomy of the left atrium (we were not sure that LAA was hosting the focus of the tachycardia). Transeptal puncture was performed and the geometry of the left atrium, of the pulmonary veins and of the LAA was reconstructed by the roving ablation catheter by means of the NavX Fusion tool and the cardiac CT scan previously obtained.

The left atrial activation mapping documented the neck of the LAA to host the tachycardia source (Figure 3A). A 4-mm Cryocath catheter was advanced inside the LAA and guided up to the site of the earliest activation signal. Starting of the cryomapping procedure (-30ºC) caused interruption of the tachycardia, preceded by transient slowing of the cycle length, about 7 seconds after the achievement of the threshold temperature (Figure 3B). Two consecutive 8-minute sequences of cryoablation (-80ºC) were then delivered. At the end of the procedure, a normal sinus rhythm was present (Figure 3D). No electrical isolation of the LAA was detected. No atrial ectopic rhythm was further inducible. Continuous intravenous heparin infusion, followed by oral anticoagulation therapy, was performed also in this case, as well as the one-day-after TEE control of the LAA, that resulted negative. The patient is still known to be asymptomatic and in sinus rhythm after 12 months of clinical follow up, in complete wash out from any antiarrhytmic drug (the last ECG control was performed 1 month ago).

## Discussion

To the best of our knowledge, these are the first cases available in the medical literature which document the safety and the efficacy of the cryoablation inside the LAA. RF energy represents the most commonly used energy source for catheter ablation. Despite the high success rate, RF energy produces tissue disruption, which increases the risk of perforation and thromboembolic events [3]. On the other hand, cryolesions are well delineated and homogeneous, since cryothermal energy creates minimal endothelial/endocardial disruption and preserves the underlying tissue architecture, thus reducing the probability of thromboembolic complications [8]. Finally, steam pops are relatively frequent during RF applications but obviously impossible to happen during cryothermal applications. Conventional RF procedures in the left atrium are generally performed aiming to avoid accidental LAA penetration by the ablation catheter during RF delivery, because of the higher risk to generate steam pops and perforation or local thrombosis and possible systemic embolism [3]. Left sided focal atrial tachycardia is a common form of arrhythmia, but its origin inside the LAA is uncommon [9]. Some studies report successful treatment of this arrhythmia by conventional RF ablation in the absence of any complication [10-12], even though such procedure is likely to involve a higher risk of thrombosis and embolization.

The very low thrombogenic power and the reduced risk of cardiac perforation during the cryoablation allowed us to successfully treat the arrhythmias in the absence of LAA harming or thrombi generation, as confirmed by the post-procedural TEE. The NavX mapping allows different catheter types to be monitored during their movements inside the heart; for this reason we were able to advance the cryoablator right towards the focus of the tachycardia, by the way avoiding the patients and the operators to receive high doses of fluoroscopy. We decided to perform the first ablation by using a 4-mm catheter, since it was the first time we were delivering cryoablation inside the LAA and thus we wanted to be as less harmful as possible. After the success of the first procedure we decided not to change the catheter size during the second one. We obviously cannot be sure wether the use of a higher size could result in a quicker termination of the arrhythmia. These findings should encourage the use of the cryoablation in potentially high thrombogenic settings or when a higher probability of steam pops and/or perforation is present. Further investigation by randomized studies of cryo vs RF ablation in a larger case series is anyway necessary to validate this topic.
